# *Angelica sinensis* Improves Exercise Performance and Protects against Physical Fatigue in Trained Mice

**DOI:** 10.3390/molecules19043926

**Published:** 2014-03-31

**Authors:** Tzu-Shao Yeh, Chi-Chang Huang, Hsiao-Li Chuang, Mei-Chich Hsu

**Affiliations:** 1 School of Nutrition and Health Sciences, Taipei Medical University, Taipei 11031, Taiwan; 2 Graduate Institute of Sports Science, College of Exercise and Health Sciences, National Taiwan Sport University, Taoyuan 33301, Taiwan; 3 National Laboratory Animal Center, National Applied Research Laboratories, Taipei 11529, Taiwan; 4 Department of Sports Medicine, Kaohsiung Medical University, Kaohsiung 80708, Taiwan

**Keywords:** Dong Quai, exhaustion, lactate, glycogen, exercise training

## Abstract

*Angelica sinensis* (AS) is a well-known medicinal herb and food material with antioxidative and multifunctional pharmacological activities. However, we lack evidence of the effect of AS on exercise performance and physical fatigue. We aimed to evaluate the potential beneficial effect of AS on ergogenic and anti-fatigue functions after physiological challenge. Male ICR strain mice were randomly assigned to four groups (*n* = 10 per group) for treatment: (1) sedentary control and vehicle treatment (vehicle control); (2) exercise training with vehicle treatment (exercise control); (3) exercise training with AS treatment at 0.41 g/kg/day (Ex-AS1); and (4) 2.05 g/kg/day (Ex-AS5); both the vehicle and AS were orally administered for 6 weeks. Exercise performance and anti-fatigue function were evaluated by forelimb grip strength, exhaustive swimming time, and levels of serum lactate, ammonia, glucose, and creatine kinase (CK) after a 15-min swimming exercise. Trend analysis revealed that AS treatments significantly increased endurance swimming time and blood glucose level, and decreased serum lactate, ammonia and CK levels. Liver and muscle glycogen contents were higher for Ex-AS1 and Ex-AS5 groups than the exercise control. Therefore, AS supplementation improved exercise performance and had anti-fatigue properties in mice and may be an effective ergogenic aid in exercise training.

## 1. Introduction

Fatigue is explained as physical and/or mental weariness that has negative impacts on exercise intensity, work performance, family life, and social relationships [1]. Physical fatigue is also called peripheral fatigue, which can affects the biochemical transduction within the exercising muscle cells, due to cytosolic accumulation of inorganic phosphate, protons and lactate [2,3,4]. Strenuous exercise has been demonstrated to increase free radicals overproduction, which leads to fatigue and tissues damage [5,6]. Several studies have shown that supplements can reduce exercise-induced physical fatigue [7,8,9,10]. We research in specific nutrients or herbal supplements to take it as ergogenic aid to reduce metabolite production and improve energy utilization during exercise.

The dried root of *Angelica sinensis* (AS), a well-known Chinese herb, commonly known as Chinese angelica or Dong Quai, is widely used in traditional Chinese medicine for gynecological ailments [11,12]. The main chemical constituents of AS roots are ferulic acid, ligustilide, polysaccharides, saponins, flavonoids, amino acid, nicotinic acid, uracil, and adenine [13]. Ferulic acid has various pharmacological activities and is used as a quality-control marker component of AS in the Chinese Pharmacopoeia [13].

In recent years, *in vitro* and *in vivo* studies elucidated the various pharmacological activities of AS stimulating the secretion of erythropoietin [14], preventing cardiovascular diseases [15], and having antioxidative [11], immune modulation [16], anti-virus [17] activities. In addition, Danggui Buxue Tang (DBT; a Chinese herbal decoction that is a simple combination of two herbs AS and *Astragalus membranaceus*) can ameliorate exercise-induced oxidative stress by modulating numerous immune system components [18]. However, relatively few studies have directly addressed the possible anti-fatigue function of AS.

Pharmacognosy and food components are important resources to be used as daily supplements for relieving fatigue, balancing fatigue-related metabolites, improving exercise performance, and health management in athletes [19,20]. For a long time, AS has been considered a potent remedy for regulating the body balance, with few adverse effects, but we have little scientific evidence for its action. AS may be an anti-fatigue herbal supplement candidate. We conducted this study to evaluate the potential ergogenic and anti-fatigue effects of AS *in vivo*.

## 2. Results and Discussion

### 2.1. Body Weight and Other Metabolism-Related Organ Weights

**Figure 1:**
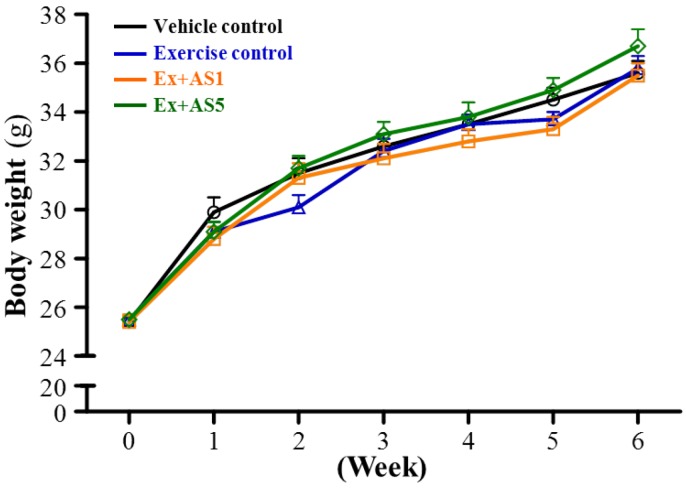
Effect of AS supplementation on the changes of body weight. Data are mean ± SEM for *n* = 10 mice per group.

Male ICR strain mice were randomly assigned to four groups (*n* = 10 per group) for treatment: (1) sedentary control and vehicle treatment (vehicle control); (2) exercise training with vehicle treatment (exercise control); (3) exercise training with AS treatment at 0.41 g/kg/day (Ex-AS1); and (4) 2.05 g/kg/day (Ex-AS5). After 6-week aerobic exercise training or AS supplementation, the final body weight at the end of the study was 35.6 ± 0.5, 35.8 ± 0.5, 35.5 ± 0.5 and 36.7 ± 0.7 g, respectively, with no significant difference between four groups (Figure 1). In addition, the four groups did not differ in absolute or relative tissue mass of liver, kidney, both gastrocnemius and soleus muscles in the back part of the lower legs, epididymal fat pad and brown adipose tissue (Table 1).

**Table 1:** General characteristics of the experimental groups.

Characteristic	Vehicle Control				Exercise Control				Ex-AS1				Ex-AS5			
***Weight (g)***																
Liver	2.16	±	0.03		2.14	±	0.03		2.11	±	0.04		2.14	±	0.04	
Kidney	0.61	±	0.01		0.65	±	0.02		0.64	±	0.01		0.66	±	0.01	
EFP	0.52	±	0.03		0.47	±	0.02		0.44	±	0.04		0.46	±	0.04	
Muscle	0.36	±	0.01		0.36	±	0.01		0.36	±	0.01		0.38	±	0.00	
BAT	0.13	±	0.01		0.15	±	0.01		0.15	±	0.01		0.13	±	0.01	
***Relative weight (%)***																
Liver	6.07	±	0.07		5.97	±	0.07		5.93	±	0.08		5.84	±	0.13	
Kidney	1.72	±	0.03		1.81	±	0.04		1.81	±	0.03		1.81	±	0.03	
EFP	1.45	±	0.06		1.30	±	0.07		1.25	±	0.10		1.25	±	0.10	
Muscle	1.01	±	0.02		1.02	±	0.02		1.02	±	0.03		1.03	±	0.02	
BAT	0.37	±	0.01		0.42	±	0.02		0.41	±	0.02		0.37	±	0.02	

Data are mean ± SEM for 10 mice in each group. EFP: epididymal fat pads; BAT: brown adipose tissue. Muscle mass includes both gastrocnemius and soleus muscles in the back part of the lower legs.

### 2.2. Effect of AS on Forelimb Grip Strength

**Figure 2:**
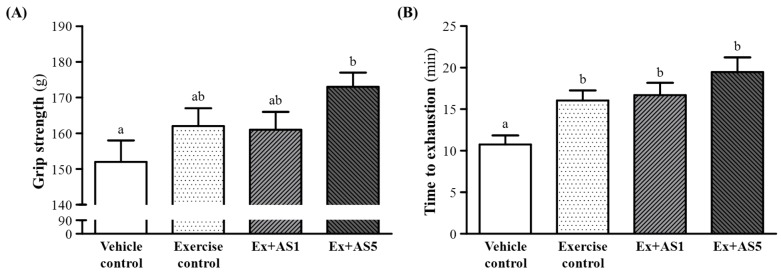
Effect of AS supplementation on forelimb grip strength (**A**) and endurance swimming time (**B**). Data are mean ± SEM of 10 mice in each group by one-way ANOVA. Different letters (a and b) indicate significant difference at *p* < 0.05.

The forelimb grip strength of mice was increased from vehicle control to Ex-AS5 group (Figure 2A). On trend analysis, AS treatments showed a slight increased grip strength (*p* = 0.0616). A regular training program is needed for grip strength increase, and AS treatment benefited grip strength with training intervention conditions. AS may improve forelimb grip strength under a training protocol.

### 2.3. Effect of AS on Exercise Performance in a Weight-Loaded Swimming Test

Exercise endurance is an important variable in evaluating anti-fatigue treatment. Exercise endurance times with a swimming test were increased from vehicle control to exercise control, Ex-AS1, and Ex-AS5 groups (Figure 2B). At the higher AS doses, exercise performance was significantly longer, by 1.49- (*p* = 0.0116), 1.55- (*p* = 0.0052) and 1.81-fold (*p* = 0.0001), with exercise control, Ex-AS1 and Ex-AS5 groups, respectively, as compared with vehicle control. However, swimming times did not differ among the exercise control, Ex-AS1, and Ex-AS5 groups. On trend analysis, AS dose-dependently increased exercise performance with training intervention (*p* = 0.0486). Therefore, AS supplementation exhibit a synergistic ergogenic effect of aerobic exercise training.

### 2.4. Effect of Exercise Combined with AS Supplementation on Mouse Serum Lactate, Ammonia, and Glucose Levels and CK Activity after Acute Exercise Challenge

**Figure 3:**
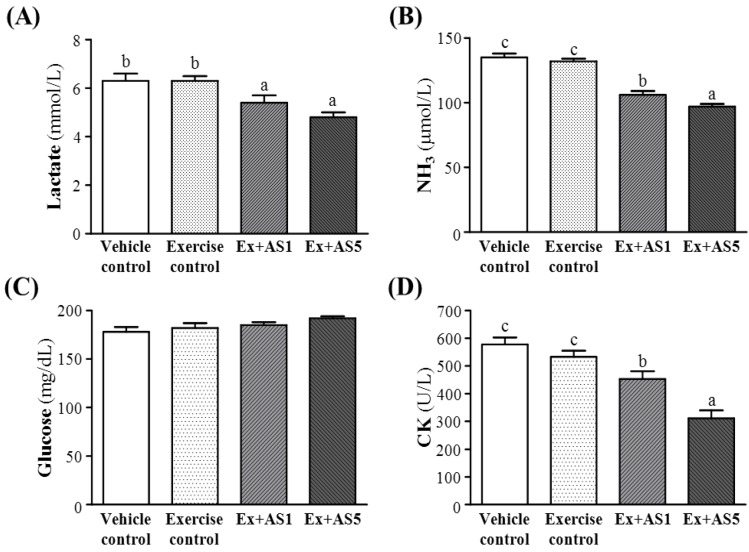
Effect of AS supplementation on serum lactate (**A**), ammonia (**B**), and glucose (**C**) levels and CK (**D**) activity immediately after 15-min swimming test without weight loading. Data are mean ± SEM of 10 mice in each group by one-way ANOVA. Different letters (a, b and c) indicate significant difference at *p* < 0.05.

Muscle fatigue after exercise can be evaluated by important biochemical indicators, including lactate, ammonia, glucose, and CK levels after exercise [21]. Lactate levels were decreased from vehicle control, exercise control to Ex-AS1 and Ex-AS5 groups (Figure 3A). Serum ammonia levels were decreased (Figure 3B), and serum CK activities, a muscle-damage marker, were decreased from vehicle control, exercise control to Ex-AS1 and Ex-AS5 groups (Figure 3D). Trend analysis revealed that AS treatments dose-dependently increased blood glucose content (*p* = 0.0234) (Figure 3C) and decreased serum lactate and ammonia and CK levels with training intervention (all *p* < 0.0001).

### 2.5. Effect of AS Supplementation on Hepatic and Muscle Glycogen Level

**Figure 4:**
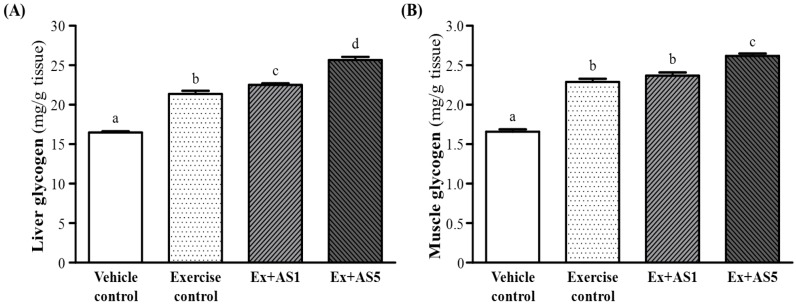
Effect of AS supplementation on hepatic glycogen (**A**) and muscle glycogen (**B**) level. Data are mean ± SEM of 10 mice in each group by one-way ANOVA. Different letters (a, b, c and d) indicate significant difference at *p* < 0.05.

Hepatic glycogen levels were increased from the vehicle control, exercise control to Ex-AS1 and Ex-AS5 groups (Figure 4A). Muscle glycogen levels were increased (Figure 4B). Trend analysis revealed that AS treatments dose-dependently increased hepatic and muscle glycogen levels with training intervention (both *p* < 0.0001).

### 2.6. Effect of AS Supplementation on Biochemical Analyses at the End of the Experiment

We examined whether AS treatment for 6 weeks could have a negative effect on healthy mice. We examined the general clinical biochemical markers in AS-treated mice (Table 2) and found no indication of a deleterious effect with AS treatment. On exercise training with continuous AS supplementation for 6 weeks, triglycerides (TG) level significantly decreased by about 36% (*p* < 0.05) with Ex-AS1 as compared with vehicle control. However, TG level did not differ among the vehicle control and Ex-AS5 groups. We suggest that the recommend dose of AS may exhibit a synergistic antihyperlipidemic effect of aerobic exercise training according to our *in vivo* data.

**Table 2:** Biochemical analysis of AS treatment groups at the end of the experiment.

Parameter	Vehicle Control	Exercise Control	Ex-AS1	Ex-AS5
AST (U/L)	63 ± 3	69 ± 4	66 ± 3	67 ± 3
ALT (U/L)	42 ± 3 ^a^	54 ± 2 ^b^	47 ± 2 ^ab^	48 ± 3 ^ab^
ALP (U/L)	49 ± 3	63 ± 6	59 ± 3	54 ± 2
LDH (U/L)	301 ± 19	273 ± 23	304 ± 15	297 ± 20
Albumin (g/dL)	3.6 ± 0.1	3.8 ± 0.1	3.7 ± 0.1	3.7 ± 0.0
TBIL (μg/dL)	0.19 ± 0.03	0.22 ± 0.03	0.23 ± 0.03	0.20 ± 0.02
TP (g/dL)	4.7 ± 0.1	4.6 ± 0.1	4.7 ± 0.1	4.8 ± 0.1
BUN (mg/dL)	23.4 ± 0.8	23.1 ± 1.0	23.8 ± 1.2	21.9 ± 0.8
Creatinine (mg/dL)	0.13 ± 0.01	0.12 ± 0.01	0.14 ± 0.01	0.15 ± 0.01
UA (mg/dL)	1.43 ± 0.11 ^b^	0.83 ± 0.05 ^a^	1.45 ± 0.07 ^b^	1.10 ± 0.07 ^ab^
TG (mg/dL)	228 ± 21 ^b^	184 ± 20 ^ab^	147 ± 8 ^a^	164 ± 11 ^ab^
TC (mg/dL)	111 ± 4	104 ± 4	122 ± 5	122 ± 5
Glucose (mg/dL)	180 ± 6	182 ± 7	185 ± 6	175 ± 5

Data are mean ± SEM for 10 mice in each group. Data in the same line with different letters (a and b) significantly differ at *p* < 0.05 by one-way ANOVA. AST, aspartate aminotransferase; ALT, alanine aminotransferase; ALP, alkaline phosphatase; LDH, lactate dehydrogenase; TBIL, total bilirubin; TP, total protein; BUN, blood urea nitrogen; UA, uric acid; TG, triacylglycerol; TC, total cholesterol.

### 2.7. Effect of AS Supplementation on Histological Examinations at the End of the Experiment

**Figure 5:**
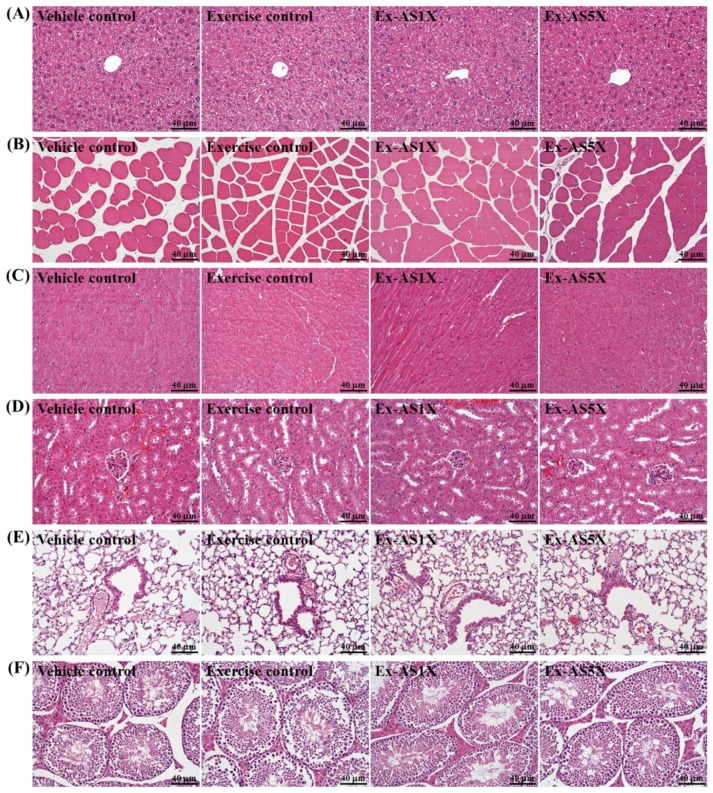
Effect of AS supplementation on the morphology of liver (**A**), skeletal muscle (**B**), heart (**C**), kidney (**D**), lungs (**E**), and testes (**F**) tissues. Specimens were photographed with a light microscope. (H&E stain, magnification: ×200, Scale bar, 40 μm).

We also examined the major organs including the liver, skeletal muscles, heart, kidney, lungs, and testes by histopathology in AS-treated mice. As shown in Figure 5, the four groups did not differ in histological observations of any of the target tissues.

### 2.8. Discussion

The effect of AS, a well-known medicinal herb and food material with antioxidative and multifunctional pharmacological activities, on exercise-induced accumulation of products of metabolism and physical toxicity has been unclear. In this study, we compared the fatigue-alleviating effects of two doses of AS and vehicle and exercise control on endurance in exercised and weight-loading mice. We found that: (1) AS supplementation potentiated the increased endurance exercise capacity with exercise training and increased hepatic and muscular glycogen content; (2) AS reduced exercise-induced accumulation of byproducts such as blood lactate and ammonia by acute exercise challenge; and (3) biochemical parameters and histopathological examination revealed no toxic effect of 6-week AS administration. AS may have ergogenic and anti-fatigue functions.

In our *in vitro* study, we found that AS treatment could promote hypertrophy in cultured skeletal myotubes, second to the *Astragalus membranaceus* extract, via the activation of phosphatidylinositol 3-kinase (PI3K)/Akt (also termed protein kinase B)/mammalian target of rapamycin (mTOR) pathway (data not shown). Therefore, we hypothesize that AS supplementation with regular aerobic exercise training may have a synergistic effect on improving exercise performance and protecting against exercise-induced fatigue. 

Energy storage and supply is another important factor related to exercise performance. With energy expenditure during exercise, physical fatigue is mainly caused by energy consumption and deficiency [22]. Catabolized fat and carbohydrates are the main sources of energy during exercise in skeletal muscles, and glycogen is the predominant source of glycolysis for energy production. Therefore, glycogen storage directly affects exercise ability [23]. In our study, liver glycogen content was significantly higher, by 1.05- and 1.20-fold (both *p* < 0.0001), with Ex-AS1 and Ex-AS5, respectively, than exercise control. In addition, muscle glycogen content was significantly higher, by 1.03- and 1.14-fold (both *p* < 0.0001), with Ex-AS1 and Ex-AS5, respectively, than vehicle control. The glycogen-sparing effect of AS could have an important survival advantage *in situ*ations requiring extended periods of prolonged exercise endurance because glycogen depletion is associated with physical exhaustion, and slower utilization of glycogen results in improved exercise endurance. As one of the sources of blood glucose, liver glycogen plays an important role in controlling the availability of cellular energy. AS supplementation may reserve glycogen or promote gluconeogenesis. Although we found no significant differences in serum glucose content among the four groups after a 15-min swim test without weight loading, blood lactate concentration was lower in the Ex-AS5 group than the exercise control. Lactic acid is produced from carbohydrate metabolism. Therefore, AS may increase muscle towards glucose oxidation for fuel usage oxidative capacity during exercise. 

AS polysaccharides, bioactive constituent of AS, consisted of rhamnose, arabinose, mannose, glucose, galactose and has immunomodulatory activity by regulating expression of Th1 and Th2 related cytokines [24]. An adequate carbohydrate availability and stable blood glucose concentration may limit stress hormone responses [25], provide glucose as energy substrate for immune cells and help to maintain immunity [26]. In addition, DBT has been reported to ameliorate the forced swimming exercise-induced chronic fatigue syndrome through immunomodulatory activities [18]. In our study, AS treatment had a significant dose-dependent effect on increasing blood glucose after a 15-min swimming test without weight-loading. These results indicate that AS may stabilize the concentration of blood glucose during exercise and avoid exercise-induced oxidative stress. 

Ferulic acid, an abundant phenolic compound, has various pharmacological activities and is used as a quality-control marker component of AS [27]. It has reported that ferulate possesses stimulatory effects that can enhance exercise endurance capacity and reduce fatigue by elevating antioxidative potentials [28]. Strenuous exercise can cause oxidative stress, which leads to an imbalance between reactive oxygen species (ROS) production and antioxidant defense. This imbalance could physically or chemically cause tissue damage and muscular cell necrosis [29]. Furthermore, reactive oxygen species (ROSs) accumulation has been suggested to be implicated in oxidative skeletal muscle fatigue [28,30]. In our study, serum CK activity was significantly lower, by 15.0% (*p* = 0.0368) and 41.7% (*p* < 0.0001), with Ex-AS1 and Ex-AS5 groups, respectively, than exercise control. We suggested that AS supplementation may act as antioxidant with increased exercise capacity and ameliorate skeletal muscle injury induced by acute exercise challenge.

The levels of serum glucose, lactate, ammonia, and free fatty acid are known to serve as indicators of accumulated fatigue and stress caused by exercise [31]. Blood lactate is the glycolysis product of carbohydrate under anaerobic conditions, and glycolysis is the main energy source for short term intensive exercise. Because the accumulation of blood lactate causes fatigue during physical exercise, rapid removal of lactate is beneficial to relieving fatigue [32]. Ammonia, a metabolite of proteins and amino acids, showed a curvilinear response to loss of velocity that was linked to neuromuscular fatigue during resistance training [33]. An increase of blood ammonia level post exercise can be managed by the use of creatine monohydrate, possibly via aerobic phosphorylation and flux through the creatine kinase system [34]. The increase of ammonia levels is related to both peripheral and central fatigue during exercise [4]. In this study, serum lactate levels were significantly lower, by 14.5% (*p* = 0.0171) and 23.6% (*p* = 0.0002), with Ex-AS1 and Ex-AS5 groups, respectively, than exercise control. Serum ammonia levels were significantly lower by 19.3% and 26.1% (both *p* < 0.0001) with Ex-AS1 and Ex-AS5, respectively, than exercise control. This result suggests that AS supplementation may reduce exercise-induced byproduct accumulation with acute exercise challenge.

Together, our results suggest that AS supplementation may ameliorate exercise-induced oxidative stress, keep blood glucose oxidation for fuel usage during exercise, and improve excretion and the elimination of the by-products of metabolism, and thereby against physical fatigue.

## 3. Experimental

### 3.1. Experimental Design

AS powdered product was a scientifically processed Chinese herbal medication purchased from Sun Ten Pharmaceutical Co. (New Taipei City, Taiwan). Male ICR mice (4 weeks old) grown under specific pathogen-free conditions were purchased from BioLASCO (Yi-Lan, Taiwan). One week of acclimation to the environment and diet was allowed before the experiment began. All animals were fed a standard laboratory diet (No. 5001; PMI Nutrition International, Brentwood, MO, USA) and distilled water *ad libitum* and housed at room temperature (23 ± 1 °C) and 50%–60% humidity with a 12-h light/dark cycle (lights on from 06:00 to 18:00). The Institutional Animal Care and Use Committee (IACUC) of National Taiwan Sport University approved all animal experiments, and this study conformed to the guidelines of protocol IACUC-10206 approved by the IACUC ethics committee.

The dose of AS for humans on a normal diet recommended by Sun Ten Pharmaceutical Co. is about 2 g per day. The mouse AS dose (0.41 g/kg) was converted from a human equivalent dose (HED) based on body surface area by the following formula from the US Food and Drug Administration: assuming a human weight of 60 kg, the HED for 2 (g) ° 60 (kg) = 0.033 × 12.3 = mouse dose of 0.41 g/kg; the conversion coefficient 12.3 was used to account for differences in body surface area between a mouse and a human as described in our recent study [35]. All animals were randomly assigned to 4 groups (*n* = 10 per group) for swimming exercise training or AS supplementation: sedentary control and vehicle treatment (vehicle control); exercise training with vehicle treatment (exercise control); exercise training with 0.41 g/kg AS (Ex-AS1) or 2.05 g/kg AS (Ex-AS5). The vehicle group received the same volume of solution equivalent to individual body weight. Both the vehicle and AS in this experiment were given orally to each animal for 6 weeks.

### 3.2. Swimming Exercise Training

Animals in the exercise control, Ex-AS1 and Ex-AS5 groups underwent an intensive aerobic swim training program as described in our recent study [8].

### 3.3. Swim to Exhaustion Exercise Test

The swim to exhaustion exercise test involved constant loads corresponding to 5% of body weight to evaluate endurance. The swimming exercise was carried out in a round tank (65 cm high, 40 cm diameter), filled with water to 45 cm depth and maintained at 30 ± 1 °C. To avoid circadian variations in physical activity, swimming exercise was performed between 07:00 and 14:00, a period when minimal variation in endurance capacity was confirmed in mice [36]. The endurance of each mouse was recorded as the time from the beginning to exhaustion, which was determined by observing loss of coordinated movements and failure to return to the surface within 7 s. Times floating, struggling, and making necessary movements were considered in the swimming duration until exhaustion and possible drowning.

### 3.4. Forelimb Grip Strength

A low-force testing system (Model-RX-5, Aikoh Engineering, Nagoya, Japan) was used to measure the forelimb grip strength of mice undergoing vehicle, exercise, and AS treatments as described in our previous studies [37,38].

### 3.5. Determination of Blood Biochemical Variables

Clinical biochemical assessment was determined by use of an autoanalyzer (Hitachi 7060, Hitachi, Tokyo, Japan). The effect of AS on serum lactate, ammonia, and glucose levels, and CK activity were evaluated post-exercise. At 1 h after the last treatment administration, mice underwent a 15-min swimming test without weight loading. After the swimming exercise, blood samples were immediately collected from the submandibular duct of pretreated mice and centrifuged at 1,500 × *g* and 4 °C for 10 min for serum preparation.

### 3.6. Tissue Glycogen Determination

The muscle and hepatic glycogen levels were measured as described in our previous studies [10,37,38].

### 3.7. Histology Staining of Tissues

Fresh liver, skeletal muscles, heart, kidney, lungs, and testes tissues were collected and fixed in 10% formalin after mice were killed. Tissues were embedded in paraffin and cut into 4-μm thick slices for morphological and pathological evaluations as described in our previous report [10,37,38]. Tissue sections were stained with hematoxylin and eosin (H&E) and examined under a light microscope equipped with a CCD camera (BX-51, Olympus, Tokyo, Japan) by a clinical pathologist.

### 3.8. Analysis of Ferulic Acid of AS by HPLC/CAD

To confirm the quality of AS, we detect the main chemical constituent of AS. The amount of ferulic acid in AS was analyzed using a high performace liquid chromatographic method [8]. Quantification was accomplished by the comparison of peak areas from the sample with that of the reference standard. The amount of ferulic acid in AS was 0.61 mg/g.

### 3.9. Statistical Analysis

All data are expressed as the mean ± SEM. Statistical differences among groups were analyzed by one-way ANOVA and the Cochran-Armitage test for dose-effect trend analysis with SPSS 14.0 (SPSS Inc., Chicago, IL, USA). In case of significant *F* ratios, Scheffe *post-hoc* tests were used to locate the differences. Statistical significance was set at *p* < 0.05.

## 4. Conclusions

AS have anti-fatigue activity by decreasing serum lactate and ammonia levels and increasing liver and muscle glycogen deposition, thereby advantaged exercise performance in mice. Although the detailed anti-fatigue mechanisms of AS remain to be elucidated, this study provides science-based evidence to support traditional claims of anti-fatigue results with AS treatment and suggests a use for AS as an ergogenic and anti-fatigue agent.
